# Electrodiagnostic approach in entrapment neuropathies of the median and ulnar nerves

**DOI:** 10.12669/pjms.313.7416

**Published:** 2015

**Authors:** Ana Maria Galamb, Ioan Dan Minea, Liliana Rogozea

**Affiliations:** 1 Dr. Ana Maria Galamb, MSc. Department of Medical and Surgical Specialities, Faculty of Medicine, Transilvania University of Brasov, Romania; 2 Dr. Ioan Dan Minea, PhD. Professor, Department of Medical and Surgical Specialities, Faculty of Medicine, Transilvania University of Brasov, Romania; 3 Dr. Liliana Rogozea, PhD. Professor, Department of Medical and Surgical Specialities, Faculty of Medicine, Transilvania University of Brasov, Romania

**Keywords:** Electrodiagnostic, Entrapment neuropathy, Median nerve, Ulnar nerve

## Abstract

**Objective:**

The present study’s aim was to analyze the late responses’ parameters in order to determine the utility of each one.

**Methods:**

The study, conducted on a total of 325 patients with entrapment neuropathy of the median nerve and 36 with entrapment neuropathy of the ulnar nerve, included the bilateral evaluation of the median and the ulnar nerve and analysis of 20 F-wave and 4 A-wave parameters.

**Results:**

The authors emphasize the necessity of bilateral examination and that of examining the ipsilateral ulnar/median nerve, such as to calculate the difference in F-wave average latency of the median/ulnar and the ipsilateral ulnar/median nerve. This was the most sensitive parameter studied, altered in more than 70% of cases, significantly in more cases than when using only the M-wave distal latency. Also there was a statistically significant correlation between patient age and F-wave latency.

**Conclusions:**

The completed research yielded the recommendation for F-wave parameter studies to include the difference in F-wave average latency of the median/ulnar and the ipsilateral ulnar/median nerve. This parameter was also included in the composite score, along with the recommendations of the American Academy of Emergency Medicine (AAEM).

## INTRODUCTION

In the case of suspected entrapment neuropathy, electrodiagnosis is part of the first line of investigations. It is a very useful diagnostic method, able to confirm the presence of compressive neuropathy, to determine its location and severity, thereby supporting case management. In addition, it can detect the presence of overlapping polyneuropathy or other compressive neuropathy or radiculopathy.

The principle that underlies the study of late responses consists in generating an excitation in the distal portion of the nerve, the impulse propagating proximally to the spinal cord, after which returning distally where can be recorded by the surface electrodes. The present study’s aim was to analyze the late response parameters in order to determine the utility of each one, using a large number of cases.

## METHODS

The electrodiagnosis was made using a Neurospectrum 4/EPM (Neurosoft, 5, Voronin str, Ivanovo, 15032, Russia). We included a total of 325 patients with entrapment neuropathy of the median nerve in the carpal tunnel (mean age: 58.52 years; standard deviation: 14.03; male/female ratio 53.85/46.15) and 36 patients with entrapment neuropathy of the ulnar nerve (mean age 49.86 years; standard deviation: 15.18; male/female ratio 33.33/66.67). The examination included the bilateral study of the median and ulnar nerves, and the assessment of 20 F-wave and 4 A-wave parameters. In some cases, the number of studied nerves and muscles was increased for an accurate diagnosis and also electromyography was performed.

### Studied parameters

The studied parameters for the F-wave were: minimum, average, maximum latency, chronodispersion and the difference between the F-wave minimum, average, maximum latency and the M-wave latency. Further studied were the proximal minimum, average, maximum velocity, tacheodispersion, the average and maximum ratio of F/M amplitudes, the percentage of high amplitude F-waves (greater than 1mV), F-wave persistence, the percentage of repetitive F-waves and the F ratio. In terms of right-left differences, we calculated: the right-left difference between the mean latencies and average velocities of the F-wave of the median/ulnar nerve and the difference in average latency of F-waves for the median/ulnar nerve and ipsilateral ulnar/median nerve. All calculations were performed bilaterally for the median and ulnar nerves.

The studied parameters for the A-wave were: amplitude, latency, latency difference between A-wave and F-wave and the calculated distance from the generating branch to the point of stimulation.

M-wave, F-wave and A-wave latencies were calculated from the deflexion of those waves, and the amplitudes were calculated peak-to-peak.

As conclusions, a composite score was formulated in which, along with the recommendations of the American Association of Electrodiagnostic Medicine (AAEM) for entrapment neuropathies, the parameters having the highest percentage of changes have been considered.

The investigation method was approved by the Ethical Committee of the Faculty of Medicine - Transilvania University of Brasov. The study was accomplished according to the WMA Declaration of Helsinki. All patients were given a detailed explanation of the study and all provided a written informed consent.

### Statistical analysis

The data collected were analyzed using Microsoft Excel 2010 for variable means, calculation of specific formulas, frequency graphs and histograms. SPSS 13.0 for Windows was used for the bivariate Pearson correlations.

## RESULTS

### I. Entrapment neuropathy of the median nerve in the carpal tunnel

Bilateral entrapment was found in 72.62% of the cases. Unilateral right upper limb involvement was present in 21.23% of the total cases. A percentage of 29.23% of the cases had also a diagnosis of polyneuropathy, entrapment neuropathy of the ulnar nerve was present at 6.77% and cervical radiculopathy at 6.15%.

**Fig.1:**
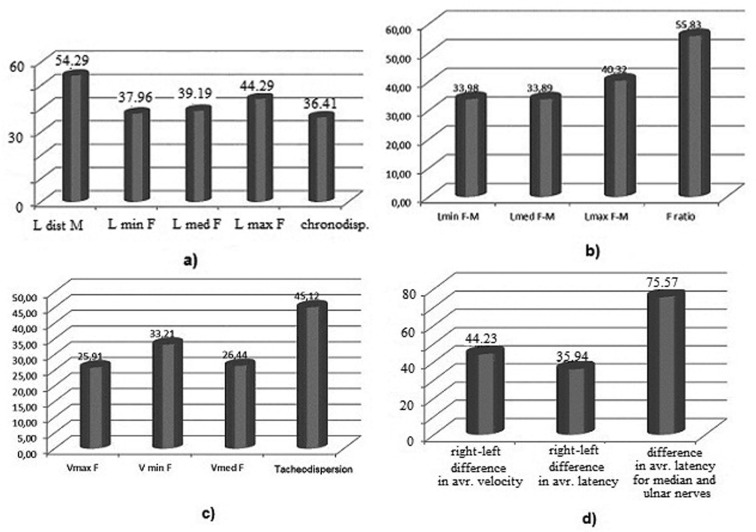
Entrapment neuropathy of the median nerve: F-wave parameters.

F-wave latencies, latency differences, chronodispersion had changes as shown in Fig.1.a, b. The percentage of alterations for F-waves with increased amplitude, and persistence were around 40% of cases. Repetitive F-waves and the average and maximum F/M-wave amplitude ratio had changes in a small number of cases. The maximum, average, minimum proximal velocity and tacheodispersion were modified as shown in Fig.1.c.

Regarding the right-left differences in average latency and velocity for the median nerve, the values did not exceed 45%. The difference of the average F-wave latency between median and ulnar nerves was altered as shown in Fig.1.d, in a large number of cases.

In only 11.08% of cases, A waves were found. The mean amplitude was 92.58 μV and the mean latency was 20.30 ms. The mean distance from where the generating branch occurred to the point of stimulation was 44.06 cm. In terms of Pearson bivariate correlations between F-wave parameters, the significantly statistical ones are presented in Table-I.

**Table-I:** Entrapment neuropathy of the median nerve: Pearson correlations between F-wave parameters.

	M latency	Min F latency	Avr F latency	Max F latency	Min F-M latency	Avr F-M latency	Max F-M latency
Age	r=0.144*	r=0.206**	r=0.170**	r=0.222**	r=0.217**	r=0.175**	r=0.191*
p=0.044	p=0.002	p=0.009	p=0.001	p=0.002	p=0.012	p=0.006	
Min F latency	r=0.574**	NA	NA	NA	NA	NA	NA
	p=0.000						
Avr F latency	r=0.604**	NA	NA	NA	NA	NA	NA
	p=0.000						
Max F latency	r=0.488**	NA	NA	NA	NA	NA	NA
	p=0.000						
Min F-M latency	NA	NA	NA	NA	NA	NA	NA
Avr F-M latency	NA	NA	NA	NA	NA	NA	NA
Chrono dispersion	r=0.021	r=0.177**	r=0.497**	r=0.183**	r=0.153*	r=0.527**	r=0.357**
	p=0.745	p=0.007	p=0.000	p=0.005	p=0.017	p=0.000	p=0.000

Key:

* = r is significant, p < 0.05,

** =r is highly significant, p < 0.01

On the other hand, F-wave velocity was positively correlated with tacheodispersion and F-ratio and negatively with the presence of F-wave blocks. Another positive correlation appeared between the F/M-wave amplitude ratio or low persistence and the presence of repetitive F-waves, and also a negative correlation with the F-ratio.

### II. Entrapment neuropathies of the ulnar nerve

About 28.40% of patients had bilateral ulnar nerve injury, 63.89% on the left limb while 91.43% of the patients had compression at the elbow.

**Fig.2:**
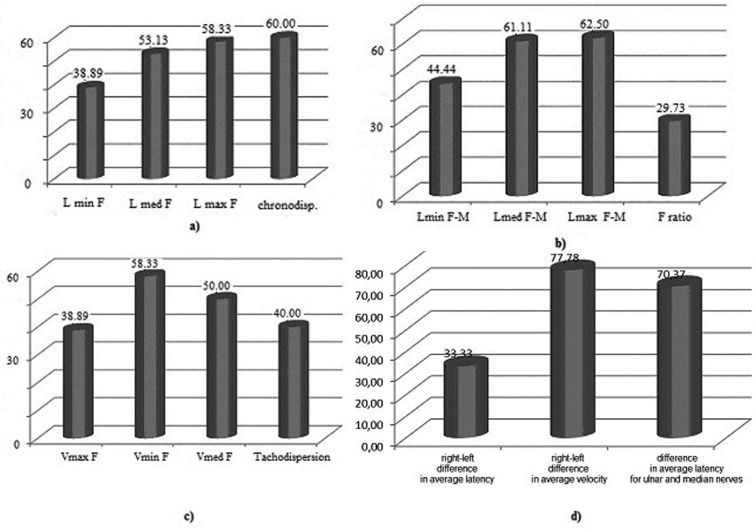
Entrapment neuropathy of the ulnar nerve: F-wave parameters.

F-wave latencies, latency differences, chronodispersion had changes as shown in Fig.2.a, b. More than half of cases had low persistence and one third presented F-waves with increased amplitude. Repetitive F-waves did not appear and the average and maximum F/M-wave amplitude ratio were altered in a small percentage of cases. The maximum, average, minimum proximal velocity and tacheodispersion were modified as shown in Fig.2.c.

The right-left differences in F-wave average latency, average velocity for ulnar nerve and average latency between the ulnar nerve and the ipsilateral median nerve were altered as shown in Fig.2.d.

A-waves occurred in 30% of cases, the average amplitude was 80.22μV, and the average latency 17.93ms. The calculated distance where the collateral branch appeared from the point of stimulation was of 41.42cm. Considering the Pearson bivariate correlations for the ulnar nerve, patient age was significantly positively correlated with the latencies of the F-waves. Another positive correlation appeared between F-wave latencies or chronodispersion and the latency difference between F and M-waves.

F-wave velocity was positively correlated with tacheodispersion and F ratio. Another correlation appeared between the F/M-wave amplitude ratio and the presence of increased amplitude F-waves with tacheodispersion or the presence of F-wave blocks (Table-II).

**Table-II:** Entrapment neuropathy of the ulnar nerve: Pearson correlations between F-wave parameters.

	Min F velocity	Avr F velocity	Max F velocity	F amplit >1mV	F/M avr
Tahodisp.	r=0.783* p=0.022	r=0.784* p=0.021	r=0.785* p=0.021	r=0.421 p=0.104	r=0.506* p=0.045
F block	r=-0.037 p=0.890	r=-0.088 p=0.745	r=-0.128 p=0.638	r=0.808* p=0.028	r=0.633* p=0.049
F ratio	r=0.739** p=0.000	r=0.681** p=0.002	r=0.664** p=0.003	r=0.143 p=0.598	r=0.008 p=0.978
F/M avr	r=-0.682* p=0.030	r=-0.655* p=0.040	r=-0.671* p=0.034	N/A	N/A

Key:

* = r is significant, p < 0.05,

** =r is highly significant, p < 0.01

## DISCUSSIONS

Concerning entrapment neuropathy of the median nerve in the carpal tunnel, the authors emphasize the necessity of bilateral examination, given the large number of cases with both right and left side injuries. Unilateral injuries occurred in most cases in the right upper limb.

The opinions concerning the role of late responses in entrapment neuropathies are very different in literature. While many authors, like Jablecki et al.^1^, believe that the study of F-waves plays no role in the diagnosis of focal lesions, other authors reinforce the role of the late responses’ parameters, like Yazdchi^2^ who studied the latencies and persistence of F-waves. In only a third of the cases we found alterations of the F-wave latencies predominantly the maximum one, contrary to Weber’s recommendations^3^ to study the minimum latency.

In half of the cases, while changes occurred in tacheodispersion and F-ratio, the most sensitive parameter was the difference between the F-wave average latency in relation to the ipsilateral ulnar nerve, which changed in nearly three quarters of the cases, confirming the necessity to examine the ipsilateral ulnar nerve. This finding is correlated to the previous studies of Kuntzer, Kim, Mehmet, Mohammed, Menkes et al.^4^-^8^

The studies of Aygul, Kim et al.^8^,^9^ reported on the role of the F/M-wave amplitude ratio and the proximal velocity in the diagnosis of carpal tunnel syndrome, but this study revealed changes in less than 30% of cases for these parameters.

A-waves were identified in only 10% of the cases having mean amplitude of approximately 90μV and an average latency of 20ms. The distance were the generating branch appeared from the stimulation point was of 44cm. Also there was a significant positive correlation between A-wave latency and the latency of the motor response.

As regards the entrapment neuropathy of the ulnar nerve, almost 90% of the cases had elbow injuries and more than half showed a left upper limb entrapment. Parameters that have experienced changes in about half the cases were F-wave average and maximum latencies, chronodispersion, latency differences compared to the M-wave, low persistence, minimum and average velocity, tacheodispersion and right-left difference in the average velocity for the ulnar nerve. Data on F-wave average latency are consistent with Weber’s study.^3^

In contrast, the difference between the average latency of the ulnar nerve and ipsilateral median nerve was altered in almost three quarters of the patients. Thus, in the case of compressive neuropathy of the ulnar nerve, examination of the ipsilateral median nerve is needed, as this difference of F-wave average latencies shows significant changes.

Anastasopoulos^10^ discussed the double crush syndrome, which in the present study was found in only 5% of the cases for compressive neuropathy of the median nerve and in 4% of the cases in the ulnar nerve.

Correlations that occurred in both types of entrapment neuropathies involved many late response parameters: Thus age is correlated with F-wave latency, entailing that a patient of older age is associated with a prolongation of the latency of these waves.

A further correlation emerged between chronodispersion and both F-wave latency and latency difference between F and M-waves, chronodispersion having greater values for increased latency. At the same time, the proximal velocity appeared related to tacheodispersion and F ratio, so a decrease in F-wave velocity will be associated with a decrease of the F ratio.

## CONCLUSIONS

The present study confirmed the utility of F wave parameters in the electrodiagnosis of entrapment neuropathies. The composite score associates to the AAEM guidelines^11^-^13^ the use of F-wave parameters as follows: for the median nerve entrapment neuropathy in the carpal tunnel, the difference between F-wave average latency in the median nerve and ipsilateral ulnar nerve; for the ulnar nerve entrapment neuropathies, the F-wave average latency difference between the ulnar nerve and the ipsilateral median nerve and the right-left difference between the average F-wave velocity for the ulnar nerve.
